# Association of SGLT2 Inhibitor Use with Glycosuria, Pyuria, Urinary Symptoms, and Significant Urine Culture Positivity in Adults with Type 2 Diabetes: A Prospective Cross-Sectional Study

**DOI:** 10.3390/medicina62091721

**Published:** 2026-09-07

**Authors:** Türkan Tüzün, Ayten Eraydın

**Affiliations:** 1 Department of Infectious Diseases and Clinical Microbiology, Faculty of Medicine, Pamukkale University, 20070 Denizli, Türkiye; 2 Division of Endocrinology and Metabolism, Department of Internal Medicine, Faculty of Medicine, Pamukkale University, 20070 Denizli, Türkiye

**Keywords:** type 2 diabetes, SGLT2 inhibitors, glycosuria, pyuria, urinary tract infection, urine culture

## Abstract

*Background and Objectives*: Sodium–glucose cotransporter 2 (SGLT2) inhibitors increase urinary glucose excretion, but the relationship between glycosuria and findings supporting urinary tract infection (UTI) remains unclear. We evaluated associations of SGLT2 inhibitor use with glycosuria, pyuria, UTI-compatible symptoms, and significant urine culture positivity in adults with type 2 diabetes. *Materials and Methods*: This prospective cross-sectional study included 251 adults who reported using every agent in their current oral antidiabetic regimen for at least three months. Blood tests, urinalysis, and midstream urine culture were obtained at a single study visit. SGLT2 inhibitor users and nonusers were compared; contaminated cultures were excluded from culture-based analyses. Multivariable logistic regression examined factors associated with glycosuria and pyuria. *Results*: Overall, 108 participants (43.0%) used an SGLT2 inhibitor (dapagliflozin, *n* = 51; empagliflozin, *n* = 57). Glycosuria was more frequent among users than nonusers (69.4% vs. 39.9%; OR = 3.43; 95% CI, 2.02–5.82; *p* < 0.001). In multivariable analysis, SGLT2 inhibitor use (adjusted OR = 3.14; 95% CI, 1.75–5.63) and each 1% increase in HbA1c (adjusted OR = 1.56; 95% CI, 1.27–1.92) were independently associated with glycosuria. Pyuria and UTI-compatible symptoms did not differ between groups. Among 217 evaluable cultures, significant culture positivity was numerically higher among users, but the estimate was imprecise (9.5% vs. 4.1%; OR = 2.45; 95% CI, 0.79–7.57; *p* = 0.110). Symptomatic culture positivity was similar (4.2% vs. 4.1%; *p* = 1.000). *Conclusions*: SGLT2 inhibitor use and higher HbA1c levels were independently associated with glycosuria. The small number of culture-positive events precludes firm conclusions about culture-based outcomes; these analyses are exploratory and do not demonstrate the presence or absence of an association. Glycosuria alone should not be interpreted as an indicator of UTI.

## 1. Introduction

Diabetes mellitus (DM) is a major public health problem with an increasing prevalence worldwide [1,2]. According to the International Diabetes Federation’s 2025 data, approximately 589 million adults aged 20–79 years are living with diabetes, and this number is projected to reach 853 million by 2050 [1]. Individuals with diabetes have increased susceptibility to infections, and urinary tract infections (UTIs) are common in this population [2,3].

Susceptibility to UTI in diabetes has been associated with glycosuria and bladder dysfunction [2]. Impaired host immune responses related to the hyperglycemic environment and increased bacterial adhesion to uroepithelial cells are also among the proposed mechanisms associated with UTI susceptibility [2,4,5]. However, the association between glycosuria and symptomatic UTI is inconsistent, and the causal role of glycosuria in the development of infection remains uncertain [2,5]. The current reference standard for UTI research considers newly developed urinary symptoms, pyuria, and quantitative urine culture findings as separate components [6]. Bacteriuria above specified quantitative thresholds, in the absence of symptoms attributable to UTI and irrespective of the presence of pyuria, is defined as asymptomatic bacteriuria [7].

Sodium–glucose cotransporter 2 (SGLT2) inhibitors reduce glucose reabsorption in the renal proximal tubule and thereby increase urinary glucose excretion [8]. The resulting pharmacological glycosuria has raised concerns about the risk of urogenital infections [5,9,10,11]. Although meta-analyses of randomized controlled trials have not demonstrated a significant increase in overall UTI risk, increases have been reported in dapagliflozin 10 mg or high-dose subgroups; however, these increases did not remain significant in analyses restricted to trials at low risk of bias [9,10,11]. Population-based observational cohorts have likewise found no increase in the risk of severe or claims-based composite UTI outcomes with SGLT2 inhibitors [12,13]. In contrast, an increased risk of genital infection has been consistently demonstrated in meta-analyses of randomized trials [9,10].

In a population-based cohort using data from the United Kingdom and Alberta, patients initiating SGLT2 inhibitors were compared with five active antidiabetic comparator groups for a composite UTI outcome based on hospital or physician-visit records [13]. In another single-center prospective cohort, the association of baseline asymptomatic pyuria and bacteriuria with three-month UTI risk was evaluated in women with type 2 diabetes who initiated an SGLT2 inhibitor [14]. In the present study, with SGLT2 inhibitor use designated as the primary exposure, we aimed to evaluate the associations of individual oral antidiabetic drug (OAD) classes and the number of different OAD classes used with glycosuria, pyuria, UTI-compatible symptoms, significant urine culture positivity, and symptomatic culture positivity in adults with type 2 diabetes.

## 2. Materials and Methods

### 2.1. Study Design and Participants

This prospective, cross-sectional, observational study was conducted at the Endocrinology and Metabolic Diseases Outpatient Clinic of Pamukkale University Hospital between January and June 2024. Patients aged 18 years or older who had type 2 diabetes mellitus, reported having used every agent in their current OAD regimen—including an SGLT2 inhibitor when applicable—for at least three months, and provided written informed consent were consecutively included. Individuals without diabetes mellitus, patients younger than 18 years, pregnant patients, patients with a urinary catheter, those who had received systemic antibiotics within the preceding three months, and those who did not consent to participate were excluded.

Eligibility with respect to treatment duration was assessed by direct questioning during the face-to-face interview. The three-month minimum applied to every agent in the current OAD regimen. Exact agent-specific durations beyond this eligibility threshold and medication doses were not prospectively recorded.

### 2.2. Determination of the Number of Participants

The study sample consisted of consecutive patients who presented to the Endocrinology and Metabolic Diseases Outpatient Clinic during the prespecified six-month enrollment period, met the inclusion and exclusion criteria, and agreed to participate. No a priori sample-size calculation specific to the primary outcome was performed. Accordingly, the primary culture-based analysis was interpreted as exploratory, with emphasis on the width of the 95% confidence interval rather than on statistical significance alone.

### 2.3. Data Collection

Demographic and clinical data were obtained through face-to-face interviews and the hospital information system. Age, sex, height, body weight, duration of diabetes mellitus, duration of OAD treatment, antidiabetic drug classes used, and use of insulin and glucagon-like peptide-1 receptor agonists were recorded. Body mass index was calculated as body weight in kilograms divided by height in meters squared.

A structured data-collection form was used to assess dysuria, urinary frequency, polyuria, hematuria, urinary incontinence, urgency, nausea, vomiting, loss of appetite, fever, chills, and rigors. Suprapubic tenderness and costovertebral angle tenderness were recorded separately as physical examination findings.

The presence of at least one symptom was defined as the presence of one or more of the following symptoms assessed on the structured form: dysuria, urinary frequency, polyuria, hematuria, urinary incontinence, urgency, nausea, vomiting, loss of appetite, fever, chills, or rigors.

Laboratory evaluation included HbA1c, serum glucose, creatinine, laboratory-reported estimated glomerular filtration rate (eGFR), C-reactive protein (CRP), complete blood count, urinalysis, and urine culture results. Urinalysis and urine culture were performed on midstream urine specimens obtained at the study visit. For blood laboratory variables, results obtained at the same study visit were used. Laboratory data were obtained from the final reports of the hospital’s biochemistry and microbiology laboratories.

Urinalysis was performed on the midstream urine specimen obtained at the study visit. Urine specimens were analyzed without centrifugation using a DIRUI FUS-2000 Urinalysis Hybrid (DIRUI Industrial Co., Ltd., Changchun, China). Urinary sediment was assessed by flow-cell digital imaging. The analyzer generated a numerical microscopic leukocyte count representing the number of white blood cells per high-power field (WBC/HPF), although the unit was not displayed on the laboratory report. The report did not provide a reference interval or a laboratory- or analyzer-specific pyuria threshold.

### 2.4. Classification of Antidiabetic Treatments

Oral antidiabetic drugs were classified as metformin, SGLT2 inhibitors, dipeptidyl peptidase-4 (DPP-4) inhibitors, sulfonylureas, thiazolidinedione/pioglitazone, and acarbose. Because the use of more than one agent was possible, each drug class was coded as a separate binary variable. Among the 108 SGLT2 inhibitor users, 51 (47.2%) received dapagliflozin and 57 (52.8%) received empagliflozin. Dose and exact agent-specific duration were not prospectively recorded. Insulin and glucagon-like peptide-1 receptor agonist use were recorded as separate variables and were not included in the number of OAD classes.

Patients were categorized into groups receiving one, two, or three or more different OAD classes. SGLT2 inhibitor use was designated as the primary exposure; analyses involving other drug classes and the number of OAD classes used were considered secondary/exploratory analyses. Treatment exposure was defined by the regimen in use at the study visit; participants were current users rather than a new-user cohort.

### 2.5. Definitions of Urinary Findings and Culture Outcomes

Glucose reported as positive by reagent-strip urinalysis was considered glycosuria. For the study analyses, pyuria was defined as a microscopic leukocyte count ≥ 5 WBC/HPF; values of 0–4 WBC/HPF were classified as negative. Numerical microscopic leukocyte counts were available for all 251 participants. The semiquantitative ‘leukocyte level’ was a leukocyte esterase result obtained with DIRUI H10-800 reagent strips for urinalysis and was categorized according to the laboratory’s analyzer settings as negative (<15), trace (±; 15 to <70), + (70 to <125), ++ (125 to <500), or +++ (≥500). No unit was assigned to these reagent-strip thresholds. The semiquantitative grades were not used for the study’s pyuria classification.

Urine culture results were classified as no growth, significant culture positivity, or contamination/mixed flora. Significant culture positivity was defined as growth of a single clinically significant bacterial pathogen at ≥10^5^ colony-forming units (CFU)/mL in a midstream urine specimen; lower colony counts were not considered positive. Growth of Candida albicans reported as a single clinically significant isolate in the microbiology laboratory’s final report was also included in the significant culture-positivity group. Specimens with growth of multiple microorganisms, particularly members of the skin or perineal flora, that were reported by the microbiology laboratory as mixed flora or contamination were considered contaminated. Isolates were identified using matrix-assisted laser desorption/ionization time-of-flight mass spectrometry. Contaminated cultures were excluded only from culture-based analyses; the patients’ non-culture clinical and laboratory data were retained in the relevant analyses.

### 2.6. Definition of UTI-Compatible Symptoms and Clinical Groups

The composite of UTI-compatible symptoms was prespecified as the presence of at least one of the following: dysuria, urinary frequency, urgency, fever, or chills/rigors. Chills and rigors were evaluated as a single combined criterion. Polyuria, nausea, vomiting, and loss of appetite alone were not considered UTI-compatible symptoms. Hematuria and urinary incontinence were reported separately as descriptive findings. Suprapubic and costovertebral angle tenderness were evaluated as physical examination findings.

Symptomatic culture positivity was defined as the coexistence of UTI-compatible symptoms and significant urine culture positivity. Patients were categorized into five groups according to UTI-compatible symptoms and culture results: (1) UTI-compatible symptoms present and significant culture positive; (2) UTI-compatible symptoms absent and significant culture positive; (3) UTI-compatible symptoms present and culture negative; (4) UTI-compatible symptoms absent and culture negative; and (5) contaminated/uninterpretable culture.

### 2.7. Study Outcomes

The primary analysis compared significant urine culture positivity between patients receiving and not receiving SGLT2 inhibitors. Secondary outcomes were glycosuria, pyuria, the presence of at least one symptom, UTI-compatible symptoms, and symptomatic culture positivity. Comparisons by the number of OAD classes used and by other OAD classes were planned as secondary/exploratory analyses.

### 2.8. Statistical Analysis

Statistical analyses were performed using IBM SPSS Statistics for macOS, version 30.0 (IBM Corp., Armonk, NY, USA). Distributional characteristics of continuous variables were evaluated using the Kolmogorov–Smirnov test. Continuous variables are presented as median and interquartile range [median (IQR)], and categorical variables as number and percentage [*n* (%)]. The Mann–Whitney U test was used to compare patients with and without significant urine culture positivity. Categorical variables were compared using Pearson’s chi-square test, Fisher’s exact test for 2 × 2 tables with inadequate expected cell frequencies, and the Fisher–Freeman–Halton exact test for 2 × 3 tables. No imputation was performed for missing data; analyses were conducted using available data. CRP data were available for 235 patients.

Glycosuria, pyuria, the presence of at least one symptom, and UTI-compatible symptoms were compared between SGLT2 inhibitor users and nonusers in the entire study population (*n* = 251). Contaminated cultures were excluded from analyses of significant culture positivity and symptomatic culture positivity, and 217 patients with evaluable cultures were analyzed. The magnitude of between-group associations was reported as odds ratios (ORs) with 95% confidence intervals (CIs).

Categorical outcomes across groups receiving one, two, or three or more OAD classes were evaluated using appropriate overall comparison tests, and linear trends between the number of OAD classes and outcomes were examined using the linear-by-linear association test. Multivariable logistic regression analyses were performed to identify factors independently associated with glycosuria and pyuria. Age, sex, SGLT2 inhibitor use, HbA1c, eGFR, and duration of diabetes mellitus were prespecified for inclusion in the regression models. Because the numbers of significant culture-positivity and symptomatic culture-positivity events were low, multivariable regression models were not constructed for these outcomes to avoid overfitting. For the 25 exploratory comparisons involving five non-SGLT2 OAD classes and five outcomes, raw Fisher’s exact *p* values were adjusted using the Benjamini–Hochberg false discovery rate (FDR) procedure; q < 0.05 was considered statistically significant. Regression results are presented as ORs with 95% CIs. Model fit was evaluated using the Hosmer–Lemeshow test, and explanatory power was assessed using Nagelkerke R^2^. All other tests were two-sided, with *p* < 0.05 denoting statistical significance. To evaluate the effect of the single Candida albicans case on culture-based outcomes, a sensitivity analysis excluding this patient was performed for significant bacterial culture positivity and symptomatic bacterial culture positivity.

### 2.9. Ethics Committee Approval

The study was approved by the Pamukkale University Non-Interventional Clinical Research Ethics Committee at meeting No. 04 held on 28 February 2023 (official letter dated 10 March 2023; No. E-60116787-020-342647). The study was conducted in accordance with the principles of the Declaration of Helsinki, and written informed consent was obtained from all participants. Reporting followed the STROBE recommendations [15].

## 3. Results

### 3.1. Participant Flow and Baseline Characteristics

**Figure 1:**
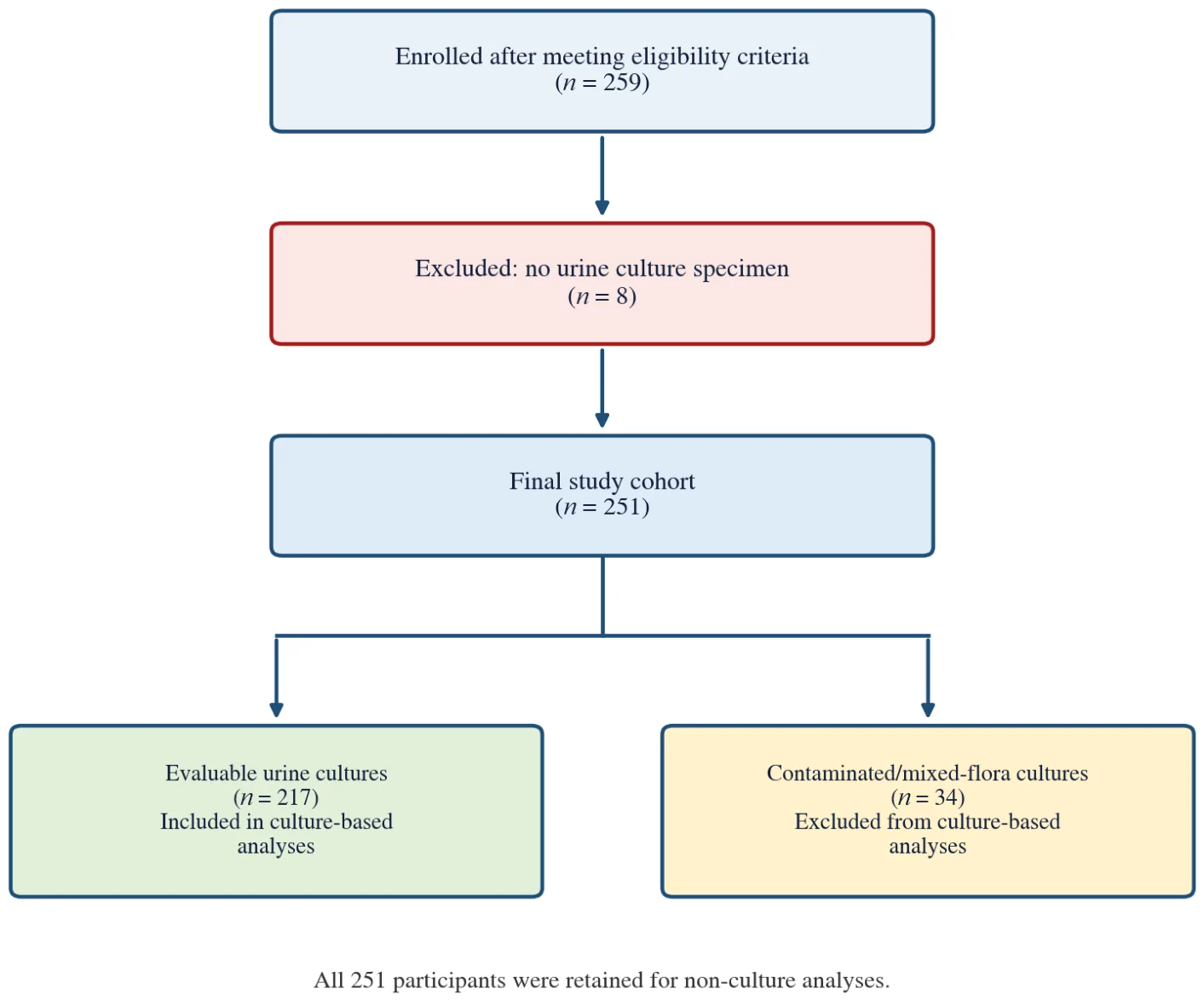
STROBE flow diagram of participant enrollment and analytic cohorts.

During the six-month study period, 259 patients who met the eligibility criteria and consented to participate were enrolled. Eight patients without a urine culture specimen were excluded, and the final analyses included 251 patients. Thirty-four patients with contamination or mixed flora were excluded only from culture-based analyses; culture-based analyses included 217 patients with evaluable cultures. The participant flow is shown in Figure 1.

Of the 251 participants, 179 (71.3%) were women and the median age was 55 years (IQR, 49–65). Overall, 108 patients (43.0%) were current SGLT2 inhibitor users; 51 (47.2%) received dapagliflozin and 57 (52.8%) received empagliflozin. Compared with nonusers, SGLT2 inhibitor users were older (median, 58 vs. 54 years; *p* = 0.019), had a higher BMI (32.7 vs. 30.4 kg/m^2^; *p* < 0.001), higher HbA1c (7.2% vs. 6.4%; *p* < 0.001), higher serum glucose (133.5 vs. 114 mg/dL; *p* = 0.010), longer diabetes duration (*p* = 0.004), and a greater number of OAD classes (median, 2 vs. 1; *p* < 0.001). Sex distribution, insulin use, creatinine, and eGFR did not differ significantly between the groups. These between-group differences underscore the potential for confounding by indication and treatment intensity (Table 1).

Glycosuria was detected in 132 patients (52.6%), pyuria in 63 (25.1%), UTI-compatible symptoms in 110 (43.8%), and at least one symptom in 121 (48.2%). Urine culture showed no growth in 203 patients (80.9%), significant growth in 14 (5.6%), and contamination/mixed flora in 34 (13.5%). UTI-compatible symptoms and significant culture positivity coexisted in nine patients (3.6%) (Supplementary Table S1).

### 3.2. Comparison of Patients with Positive and Negative Cultures

Patients with significant culture positivity had higher CRP levels and urinary erythrocyte and leukocyte counts, and more frequently had suprapubic tenderness, pyuria, at least one symptom, and dysuria. Between-group differences were statistically significant for CRP level (*p* = 0.009), urinary erythrocyte count (*p* = 0.002), urinary leukocyte count (*p* < 0.001), suprapubic tenderness (*p* < 0.001), pyuria (*p* < 0.001), the presence of at least one symptom (*p* = 0.018), and dysuria (*p* = 0.002). UTI-compatible symptoms were more frequent among culture-positive patients, but the between-group difference did not reach statistical significance (64.3% vs. 41.4%; *p* = 0.094) (Table 2).

Among the 14 patients with significant culture positivity, *Escherichia coli* was isolated in 10 (71.4%), *Klebsiella* spp. in three (21.4%), and Candida albicans in one (7.1%) (Supplementary Table S3).

### 3.3. Urinary Outcomes According to SGLT2 Inhibitor Use

Glycosuria was significantly more frequent among SGLT2 inhibitor users than among nonusers (69.4% vs. 39.9%; OR = 3.43; 95% CI, 2.02–5.82; *p* < 0.001). No statistically significant between-group differences were observed for pyuria (24.1% vs. 25.9%; OR = 0.91; *p* = 0.745), the presence of at least one symptom (43.5% vs. 51.7%; OR = 0.72; *p* = 0.196), or UTI-compatible symptoms (38.0% vs. 48.3%; OR = 0.66; *p* = 0.104).

Among patients with evaluable cultures, significant culture positivity was numerically higher among SGLT2 inhibitor users (9.5% vs. 4.1%), but the estimate was imprecise and the difference did not reach statistical significance (OR = 2.45; 95% CI, 0.79–7.57; *p* = 0.110). This exploratory result should not be interpreted as evidence that SGLT2 inhibitor use is unrelated to culture positivity. Symptomatic culture positivity occurred in 4.2% and 4.1% of patients, respectively; this estimate was likewise imprecise (OR = 1.03; 95% CI, 0.27–3.94; *p* = 1.000) (Table 3). After exclusion of the single Candida albicans case, significant bacterial culture positivity was observed in 8.5% (8/94) of SGLT2 inhibitor users and 4.1% (5/122) of nonusers (OR = 2.18; 95% CI, 0.69–6.88; *p* = 0.176). Rates of symptomatic bacterial culture positivity were 4.3% and 4.1%, respectively (OR = 1.04; 95% CI, 0.27–3.98; *p* = 1.000). These sensitivity estimates were likewise imprecise.

### 3.4. Outcomes According to the Number of OAD Classes Used

The frequency of glycosuria was 41.1%, 64.1%, and 75.0% among patients receiving one, two, and three or more OAD classes, respectively; a significant linear increase in glycosuria frequency was observed as the number of OAD classes increased (overall *p* < 0.001; *p* for trend < 0.001). There were no significant between-group differences in pyuria or the presence of at least one symptom.

The overall comparison of UTI-compatible symptom frequency according to the number of OAD classes was not statistically significant (*p* = 0.130). Nevertheless, the respective rates among patients receiving one, two, and three or more OAD classes were 48.9%, 39.7%, and 31.3%, and the trend analysis indicated a decreasing association as the number of OAD classes increased (*p* for trend = 0.044). This finding was considered secondary and exploratory. No significant differences in significant culture positivity or symptomatic culture positivity were detected across the OAD groups in either the overall comparisons or trend analyses (Table 4).

### 3.5. Multivariable Analyses

In the multivariable logistic regression model for glycosuria, SGLT2 inhibitor use (adjusted OR = 3.14; 95% CI, 1.75–5.63; *p* < 0.001) and HbA1c level (adjusted OR = 1.56; 95% CI, 1.27–1.92; *p* < 0.001) were independently associated with glycosuria. Age, sex, eGFR, and duration of diabetes mellitus showed no significant associations (Table 5).

In the pyuria model, male sex showed an independent inverse association with pyuria (adjusted OR = 0.22; 95% CI, 0.09–0.51; *p* < 0.001). SGLT2 inhibitor use, HbA1c, age, eGFR, and duration of diabetes mellitus were not independently associated with pyuria (Table 6).

### 3.6. Exploratory OAD-Class Analyses

In exploratory analyses, the raw *p* values suggested less frequent glycosuria among metformin users than among nonusers (49.3% vs. 67.4%; raw *p* = 0.033) and more frequent glycosuria among DPP-4 inhibitor users than among nonusers (67.7% vs. 47.6%; raw *p* = 0.008). Neither comparison remained statistically significant after Benjamini–Hochberg FDR correction (q = 0.415 and q = 0.201, respectively). No other OAD-class comparison was significant after FDR correction. Given the very small thiazolidinedione (*n* = 3) and acarbose (*n* = 2) groups, all class-specific results are descriptive and hypothesis-generating (Supplementary Table S2).

## 4. Discussion

In this prospective cross-sectional study of adults with type 2 diabetes, our findings support an association between SGLT2 inhibitor use and glycosuria; SGLT2 inhibitor use and HbA1c remained independently associated with glycosuria in multivariable analysis. By contrast, the culture-based analyses contained few events and yielded wide confidence intervals; they therefore do not establish the presence or absence of an association between SGLT2 inhibitor use and UTI-related outcomes. Glycosuria should consequently be interpreted in the context of antidiabetic treatment and glycemic control rather than as an indicator of UTI by itself.

SGLT2 inhibitors reduce glucose reabsorption in the renal proximal tubule and thereby increase urinary glucose excretion [8]. The potential contribution of this pharmacological glycosuria to the risk of genital and urinary infections remains clinically debated [5]. Consistent with this mechanism, glycosuria was more frequent among SGLT2 inhibitor users in our study, and SGLT2 inhibitor use remained independently associated with glycosuria in the multivariable analysis. Monobe et al. reported that mean blood glucose was independently associated with 24-h urinary glucose excretion [16]. Cui et al. found that HbA1c was independently associated with the renal glucose threshold and showed the strongest association among the variables examined [17]. The independent association observed between HbA1c and glycosuria in our study suggests that overall glycemic exposure, in addition to the pharmacological effect of SGLT2 inhibitors, may contribute to urinary glucose excretion.

Meta-analyses of randomized controlled trials have not demonstrated that SGLT2 inhibitors increase UTI risk across the class [9,10]. Similarly, most comparisons in a network meta-analysis showed no significant increase in risk [11]. However, drug- and dose-specific analyses identified a signal of increased UTI risk with dapagliflozin 10 mg/day and high-dose dapagliflozin (≥10 mg) [10,11]. Likewise, large population-based cohort studies found that initiation of an SGLT2 inhibitor was not associated with an increased risk of overall or severe UTI compared with other glucose-lowering therapies [12,13]. In our study, no statistically significant associations were identified between SGLT2 inhibitor use and pyuria, UTI-compatible symptoms, significant culture positivity, or symptomatic culture positivity. Because our culture- and symptom-based outcomes do not correspond exactly to the clinical UTI or severe UTI definitions used in previous studies, these comparisons should be interpreted cautiously.

In a propensity score-matched cohort based on administrative health records from Japan, Imai et al. reported that SGLT2 inhibitor use was associated with a lower risk of UTI but a higher risk of genital bacterial infection compared with DPP-4 inhibitor use [18]. Similarly, in a Danish cohort including 52,414 SGLT2 inhibitor users and 27,023 glucagon-like peptide-1 receptor agonist users, initiation of SGLT2 inhibitor therapy was not associated with an increased risk of UTI but was associated with a higher risk of genital tract infection [19]. A recent meta-analysis of 11 cohort studies including 679,617 patients found no association between SGLT2 inhibitor use and an increased risk of severe UTI [20]. The risk was lower than that observed with comparator groups combining other glucose-lowering drugs and with DPP-4 inhibitors, and was similar to that observed with glucagon-like peptide-1 receptor agonists [20]. Akkuş et al. reported that baseline asymptomatic pyuria or bacteriuria was not significantly associated with UTI development during three months of follow-up among women with type 2 diabetes initiating SGLT2 inhibitor therapy [14]. Taken together, these findings indicate that the increase in genital infection risk with SGLT2 inhibitors has been demonstrated more consistently than an increase in UTI risk [9,10,18,19]. However, because genital infections were not evaluated in our study, no direct inference can be made regarding this outcome.

Pyuria or bacteriuria alone is insufficient for diagnosing symptomatic UTI in the absence of UTI-compatible symptoms [7]. The multidisciplinary reference standard developed for UTI research considers newly developed urinary symptoms, different levels of pyuria, and culture findings together [6]. Although our definitions do not correspond exactly to this standard, pyuria, urinary symptoms, and significant culture positivity were examined as separate outcomes, and cases in which UTI-compatible symptoms coexisted with significant culture positivity were evaluated separately as symptomatic culture positivity. This approach enabled us to evaluate glycosuria separately from the clinical and microbiological findings supporting UTI.

Only 14 patients had significant culture positivity and only nine had symptomatic culture positivity. The resulting wide confidence intervals indicate limited statistical precision and inadequate power to exclude clinically meaningful associations. Culture-based findings should therefore be considered exploratory; a nonsignificant *p* value in this setting does not demonstrate equivalence or absence of effect.

Among patients with significant culture positivity, suprapubic tenderness, dysuria, and pyuria were more frequent, and CRP levels and urinary erythrocyte and leukocyte counts were higher. These observations are consistent with current clinical and research frameworks emphasizing that bacteriuria may occur without urinary symptoms and that symptoms, pyuria, and culture findings should be considered together when evaluating UTI [6,7]. *Escherichia coli* was the most frequently isolated microorganism in culture-positive specimens, consistent with the literature identifying it as a leading uropathogen in patients with diabetes [2]. After exclusion of the single Candida albicans case, neither the direction nor the statistical interpretation of the findings for significant bacterial culture positivity and symptomatic bacterial culture positivity changed.

In the multivariable analysis, male sex was independently and inversely associated with pyuria, indicating a higher probability of pyuria among women. Nevertheless, this secondary finding should be interpreted cautiously because the influence of unmeasured confounding cannot be excluded.

In our study, glycosuria frequency increased significantly and linearly with the number of OAD classes used, whereas no significant linear trend was observed for pyuria or culture-based outcomes. Current American Diabetes Association recommendations advise that treatment modification, including intensification, should not be delayed when individualized treatment goals are not met [21]. In a recent systematic review and meta-analysis, oral quadruple-combination therapy was considered an effective and safe option for patients with type 2 diabetes whose glycemic control remained inadequate with triple oral therapy [22]. However, owing to the cross-sectional design of our study, the association between the number of OAD classes and glycosuria cannot be interpreted as a direct effect of the number of OAD classes used. Because this association was not evaluated in a separate multivariable model, it may have been confounded by treatment composition—particularly SGLT2 inhibitor use—and glycemic status. Indeed, in our multivariable model, SGLT2 inhibitor use and HbA1c level were independently associated with glycosuria.

Although a decreasing trend in UTI-compatible symptom frequency was observed as the number of OAD classes increased, this finding should be considered exploratory because the overall group comparison was not significant and no similar trend was observed in culture-based outcomes. Similarly, nominal associations of metformin and DPP-4 inhibitor use with glycosuria did not remain statistically significant after FDR correction. The extremely small thiazolidinedione and acarbose groups make their estimates particularly unstable. These analyses do not support drug-class-specific effects and should be regarded only as hypothesis-generating.

The strengths of our study include prospective data collection, structured assessment of urinary symptoms, and examination of glycosuria, pyuria, urinary symptoms, and culture results as separate outcomes. Separate evaluation of cases in which UTI-compatible symptoms coexisted with significant culture positivity, together with exclusion of contaminated cultures from culture-based analyses, enabled pharmacological glycosuria to be assessed separately from the clinical and microbiological findings supporting UTI. In addition, examining factors associated with glycosuria and pyuria in multivariable models allowed several prespecified potential confounders to be considered.

Nevertheless, the single-center, single-visit cross-sectional design limits temporal and causal inference and generalizability. It cannot establish whether drug exposure preceded urinary findings or distinguish transient asymptomatic bacteriuria from the subsequent development of symptomatic UTI. No sample-size calculation specific to the primary outcome was performed, and the low number of culture-based events limited statistical power and the precision of the estimates. Multivariable models for culture-based outcomes could not be constructed without substantial overfitting; residual and unmeasured confounding therefore remain possible. The study also used a prevalent-user rather than a new-user design. Patients who discontinued an SGLT2 inhibitor after an early urinary adverse event may consequently be underrepresented, introducing survivor/prevalent-user bias. Although Table 1 now documents differences between exposure groups, confounding by indication and treatment intensity cannot be eliminated. A history of recurrent UTI, diabetic neurogenic bladder dysfunction, and post-void residual urine volume were not systematically collected and could not be included in adjusted analyses. In addition, antidiabetic drug doses, exact class-specific durations, and adherence were not prospectively recorded, although all agents in the current regimen had been used for at least three months. The inclusion of fever and chills/rigors in the UTI-compatible symptom composite may have reduced its specificity. The predominance of women and exclusion of patients who had used systemic antibiotics within the preceding three months further limit generalizability. Finally, the exploratory analyses of other OAD classes remained vulnerable to random error despite FDR correction, particularly because the thiazolidinedione and acarbose groups were extremely small.

## 5. Conclusions

In adults with type 2 diabetes, SGLT2 inhibitor use and higher HbA1c levels were independently associated with glycosuria. This was the principal finding supported by the data. No statistically significant between-group differences were observed in pyuria or UTI-compatible symptoms. Culture-based analyses were limited by the small number of events and wide confidence intervals and should therefore be considered exploratory. Glycosuria should not be used as a stand-alone indicator of UTI and should instead be evaluated alongside symptoms, pyuria, culture findings, antidiabetic treatment, and glycemic control.

## Figures and Tables

**Table 1:** Baseline demographic, clinical, laboratory, and treatment characteristics according to SGLT2 inhibitor use.

Characteristic	Overall (*n* = 251)	No SGLT2 Inhibitor (*n* = 143)	SGLT2 Inhibitor Use (*n* = 108)	*p*-Value
**Demographic and clinical characteristics**				
Sex				0.774
Female	179 (71.3)	103 (72.0)	76 (70.4)	
Male	72 (28.7)	40 (28.0)	32 (29.6)	
Age (years)	55 (49–65)	54 (46–64)	58 (52–65)	0.019
BMI (kg/m^2^)	31.3 (27.7–35.2)	30.4 (26.5–34.1)	32.7 (28.9–37.3)	<0.001
Duration of diabetes				0.004
3–12 months	31 (12.4)	21 (14.7)	10 (9.3)	
>1–5 years	66 (26.3)	47 (32.9)	19 (17.6)	
>5 years	154 (61.4)	75 (52.4)	79 (73.1)	
**Antidiabetic treatment**				
Metformin	205 (81.7)	127 (88.8)	78 (72.2)	<0.001
DPP-4 inhibitor	62 (24.7)	28 (19.6)	34 (31.5)	0.030
Sulfonylurea	14 (5.6)	6 (4.2)	8 (7.4)	0.272
Thiazolidinedione	3 (1.2)	2 (1.4)	1 (0.9)	1.000
Acarbose	2 (0.8)	2 (1.4)	0 (0.0)	0.508
Insulin	46 (18.3)	28 (19.6)	18 (16.7)	0.555
GLP-1 receptor agonist	20 (8.0)	13 (9.1)	7 (6.5)	0.450
Reported duration of OAD treatment				0.004
3–12 months	51 (20.3)	36 (25.2)	15 (13.9)	
>1–5 years	85 (33.9)	54 (37.8)	31 (28.7)	
>5 years	115 (45.8)	53 (37.1)	62 (57.4)	
Number of OAD classes used	1 (1–2)	1 (1–1)	2 (2–3)	<0.001
OAD-class count category				<0.001
1 OAD class	141 (56.2)	122 (85.3)	19 (17.6)	
2 OAD classes	78 (31.1)	20 (14.0)	58 (53.7)	
≥3 OAD classes	32 (12.7)	1 (0.7)	31 (28.7)	
**SGLT2 inhibitor agent among users**				
Dapagliflozin	51 (20.3)	—	51 (47.2)	—
Empagliflozin	57 (22.7)	—	57 (52.8)	—
**Laboratory characteristics**				
HbA1c (%)	6.7 (6.1–8.2)	6.4 (5.9–7.9)	7.2 (6.5–8.7)	<0.001
Serum glucose (mg/dL)	124 (104–162)	114 (102–157)	133.5 (110–167.5)	0.010
Creatinine (mg/dL)	0.77 (0.68–0.91)	0.77 (0.7–0.91)	0.79 (0.67–0.91)	0.682
eGFR (mL/min/1.73 m^2^)	91 (77–101)	91 (76–102.5)	89.5 (77–98)	0.298
Leukocyte count (10^3^/µL)	8 (6.57–9.68)	8.05 (6.72–9.75)	7.9 (6.49–9.6)	0.471
Neutrophil count (10^3^/µL)	4.46 (3.5–5.67)	4.5 (3.57–5.67)	4.3 (3.33–5.68)	0.399
Lymphocyte count (10^3^/µL)	2.36 (1.9–2.88)	2.44 (1.98–2.86)	2.34 (1.88–2.9)	0.543
CRP (mg/L), *n* = 235	2.5 (0.99–5.2)	2.47 (1.08–5.43)	2.6 (0.87–5)	0.629

Data are presented as *n* (%) or median (IQR). Percentages in the exposure columns use the respective group denominator. Continuous variables were compared using the Mann–Whitney U test; categorical variables were compared using Pearson’s chi-square test or Fisher’s exact test, as appropriate. CRP data were available for 235 patients (134 nonusers and 101 users). SGLT2 inhibitor exposure was defined by the current regimen at enrollment; every current OAD, including an SGLT2 inhibitor when applicable, was reported during the face-to-face interview to have been used for at least three months. The treatment-duration categories are not agent-specific. Agent percentages are calculated among the 108 SGLT2 inhibitor users; doses and exact agent-specific durations were not prospectively recorded. OAD, oral antidiabetic drug; SGLT2, sodium–glucose cotransporter 2; DPP-4, dipeptidyl peptidase-4; GLP-1, glucagon-like peptide-1; BMI, body mass index; eGFR, estimated glomerular filtration rate; CRP, C-reactive protein. Shaded rows indicate category or panel headings.

**Table 2:** Comparison of patients with and without significant urine culture positivity.

Variable	Culture Negative (*n* = 203)	Significant Culture Positivity (*n* = 14)	*p*-Value
Sex			0.070
Female	139 (68.5)	13 (92.9)	
Male	64 (31.5)	1 (7.1)	
Age (years)	55 (48–63)	60 (51–67)	0.293
Height (cm)	159 (154–165)	155.5 (150–160)	0.155
Weight (kg)	80 (70–92)	79.5 (68–93.5)	0.883
BMI (kg/m^2^)	30.9 (27.7–35.3)	34 (26.8–36.8)	0.476
Duration of diabetes			0.655
3–12 months	27 (13.3)	1 (7.1)	
>1–5 years	55 (27.1)	3 (21.4)	
>5 years	121 (59.6)	10 (71.4)	
SGLT2 inhibitor	86 (42.4)	9 (64.3)	0.110
Metformin	165 (81.3)	12 (85.7)	1.000
DPP-4 inhibitor	48 (23.6)	2 (14.3)	0.530
Sulfonylurea	13 (6.4)	1 (7.1)	1.000
Thiazolidinedione	3 (1.5)	0 (0)	1.000
Acarbose	2 (1)	0 (0)	1.000
Insulin	39 (19.2)	5 (35.7)	0.166
GLP-1 receptor agonist	18 (8.9)	1 (7.1)	1.000
Duration of OAD use			0.586
3–12 months	41 (20.2)	3 (21.4)	
1–5 years	70 (34.5)	3 (21.4)	
>5 years	92 (45.3)	8 (57.1)	
Suprapubic tenderness	9 (4.4)	6 (42.9)	<0.001
Costovertebral angle tenderness	12 (5.9)	1 (7.1)	0.590
Laboratory variables			
HbA1c (%)	6.6 (6.1–8.1)	7.5 (6.5–9.1)	0.246
Serum glucose (mg/dL)	122 (103–160)	140 (111–174)	0.205
Creatinine (mg/dL)	0.8 (0.7–0.9)	0.7 (0.6–1)	0.233
eGFR (mL/min/1.73 m^2^)	91 (77–101)	93.5 (73–97)	0.703
Leukocyte count (10^3^/µL)	8 (6.5–9.6)	7.8 (6.8–9.2)	0.886
Neutrophil count (10^3^/µL)	4.5 (3.5–5.7)	3.9 (3.4–5.8)	0.753
Lymphocyte count (10^3^/µL)	2.4 (1.9–2.9)	2.5 (2.2–2.8)	0.793
CRP (mg/L)	2.5 (1–5)	5.6 (2.4–25)	0.009
Urinalysis			
Urinary erythrocyte count (RBC/HPF)	1 (1–2)	6 (1–16)	0.002
Microscopic urinary leukocyte count (WBC/HPF)	1 (0–3)	15.5 (8–28)	<0.001
Pyuria	39 (19.2)	12 (85.7)	<0.001
Urine glucose grade (0–3)	1 (0–3)	0 (0–3)	0.789
Glycosuria	108 (53.2)	6 (42.9)	0.453
UTI-compatible symptoms	84 (41.4)	9 (64.3)	0.094
Presence of at least one symptom	93 (45.8)	11 (78.6)	0.018
Dysuria	36 (17.7)	8 (57.1)	0.002
Urinary frequency	62 (30.5)	3 (21.4)	0.562
Polyuria	26 (12.8)	1 (7.1)	1.000
Hematuria	4 (2)	0 (0)	1.000
Urinary incontinence	35 (17.2)	5 (35.7)	0.144
Urgency	48 (23.6)	1 (7.1)	0.200
Nausea	14 (6.9)	1 (7.1)	1.000
Vomiting	6 (3)	1 (7.1)	0.377
Loss of appetite	7 (3.4)	2 (14.3)	0.107
Fever	8 (3.9)	0 (0)	1.000
Chills	19 (9.4)	0 (0)	0.618
Rigors	24 (11.8)	1 (7.1)	1.000
Presence of chills/rigors	32 (15.8)	1 (7.1)	0.700
Number of OAD classes used	1 (1–2)	2 (1–2)	0.360
Group according to the number of OAD classes			0.551
1 OAD class	116 (57.1)	6 (42.9)	
2 OAD classes	61 (30)	6 (42.9)	
≥3 OAD classes	26 (12.8)	2 (14.3)	

Thirty-four cultures reported as contamination/mixed flora were excluded from this comparison. Continuous variables were compared using the Mann–Whitney U test; categorical variables were compared using Pearson’s chi-square test or Fisher’s exact test, as appropriate for expected cell frequencies. CRP was analyzed among patients with available data. The analyzer’s numerical microscopic leukocyte output represented WBC/HPF, although the unit was not displayed on the laboratory report. Because numerous secondary comparisons were performed, the results should be interpreted as exploratory; no adjustment for multiple comparisons was applied. Shaded rows indicate category or panel headings.

**Table 3:** Urinary findings and culture-based outcomes according to SGLT2 inhibitor use.

Variable	No SGLT2 Inhibitor	SGLT2 Inhibitor Use	OR (95% CI)	*p*-Value
Panel A. All patients (*n* = 251)	*n* = 143	*n* = 108		
Glycosuria	57 (39.9)	75 (69.4)	3.43 (2.02–5.82)	<0.001
Pyuria	37 (25.9)	26 (24.1)	0.91 (0.51–1.62)	0.745
At least one symptom	74 (51.7)	47 (43.5)	0.72 (0.43–1.19)	0.196
UTI-compatible symptoms	69 (48.3)	41 (38.0)	0.66 (0.40–1.09)	0.104
Panel B. Patients with evaluable cultures (*n* = 217)	*n* = 122	*n* = 95		
Significant culture positivity	5 (4.1)	9 (9.5)	2.45 (0.79–7.57)	0.110
Symptomatic culture positivity *	5 (4.1)	4 (4.2)	1.03 (0.27–3.94)	1.000
Panel C. Sensitivity analysis excluding the C. albicans case (*n* = 216)	*n* = 122	*n* = 94		
Significant bacterial culture positivity	5 (4.1)	8 (8.5)	2.18 (0.69–6.88)	0.176
Symptomatic bacterial culture positivity *	5 (4.1)	4 (4.3)	1.04 (0.27–3.98)	1.000

OR, odds ratio; CI, confidence interval. Panel A includes the entire study population. Panel B includes patients with evaluable cultures after exclusion of contaminated cultures. In Panel C, the single Candida albicans case—an SGLT2 inhibitor user without UTI-compatible symptoms—was excluded. At least one symptom denotes the presence of one or more symptoms assessed on the structured form. UTI-compatible symptoms were defined as the presence of at least one of dysuria, urinary frequency, urgency, fever, or chills/rigors. * Symptomatic culture positivity was defined as the coexistence of UTI-compatible symptoms and significant culture positivity. Pearson’s chi-square test was used for significant culture positivity and bacterial culture positivity in the sensitivity analysis; Fisher’s exact test was used for symptomatic culture positivity. Shaded rows indicate category or panel headings.

**Table 4:** Urinary findings and culture-based outcomes according to the number of OAD classes used.

Variable	1 OAD Class	2 OAD Classes	≥3 OAD Classes	*p*-Value	*p* for Trend
Panel A. All patients (*n* = 251)	*n* = 141	*n* = 78	*n* = 32		
Glycosuria	58 (41.1)	50 (64.1)	24 (75.0)	<0.001	<0.001
Pyuria	32 (22.7)	22 (28.2)	9 (28.1)	0.610	0.371
At least one symptom	74 (52.5)	36 (46.2)	11 (34.4)	0.164	0.063
UTI-compatible symptoms	69 (48.9)	31 (39.7)	10 (31.3)	0.130	0.044
Panel B. Patients with evaluable cultures (*n* = 217)	*n* = 122	*n* = 67	*n* = 28		
Significant culture positivity	6 (4.9)	6 (9.0)	2 (7.1)	0.529	0.423
Symptomatic culture positivity *	5 (4.1)	2 (3.0)	2 (7.1)	0.634	0.667

OAD, oral antidiabetic drug. Contaminated cultures were excluded from Panel B analyses. The Fisher–Freeman–Halton exact test was used for culture-based comparisons across the three groups. Trend *p* values were calculated using the linear-by-linear association test. * Symptomatic culture positivity was defined as the coexistence of UTI-compatible symptoms and significant culture positivity. Shaded rows indicate category or panel headings.

**Table 5:** Multivariable logistic regression analysis of factors independently associated with glycosuria (*n* = 251).

Variable	Adjusted OR (95% CI)	*p*-Value
Age (per 1-year increase)	0.978 (0.949–1.007)	0.141
Sex		
Female	Reference	-
Male	1.381 (0.712–2.677)	0.339
SGLT2 inhibitor	3.138 (1.751–5.625)	<0.001
HbA1c (per 1% increase)	1.563 (1.272–1.917)	<0.001
eGFR (per 1 mL/min/1.73 m^2^ increase)	0.995 (0.978–1.013)	0.568
Duration of diabetes		
3–12 months	Reference	-
>1–5 years	0.935 (0.354–2.470)	0.892
>5 years	1.159 (0.464–2.898)	0.752

OR, odds ratio; CI, confidence interval; eGFR, estimated glomerular filtration rate. Hosmer–Lemeshow test *p* = 0.878; Nagelkerke R^2^ = 0.279. Shaded rows indicate category or panel headings.

**Table 6:** Multivariable logistic regression analysis of factors independently associated with pyuria (*n* = 251).

Variable	Adjusted OR (95% CI)	*p*-Value
Age (per 1-year increase)	1.031 (0.998–1.065)	0.068
Sex		
Female	Reference	-
Male	0.216 (0.091–0.511)	<0.001
SGLT2 inhibitor	0.701 (0.373–1.318)	0.270
HbA1c (per 1% increase)	1.145 (0.953–1.375)	0.148
eGFR (per 1 mL/min/1.73 m^2^ increase)	0.999 (0.982–1.017)	0.943
Duration of diabetes		
3–12 months	Reference	-
>1–5 years	0.979 (0.303–3.167)	0.972
>5 years	1.371 (0.453–4.153)	0.577

OR, odds ratio; CI, confidence interval; eGFR, estimated glomerular filtration rate. Hosmer–Lemeshow test *p* = 0.288; Nagelkerke R^2^ = 0.125. Shaded rows indicate category or panel headings.

## Data Availability

The datasets generated and analyzed during the current study are not publicly available because of participant privacy and ethical restrictions. The data are available from the corresponding author upon reasonable request and subject to the necessary institutional and ethical approvals.

## Supplementary Materials

The following supporting information can be downloaded at: https://www.mdpi.com/article/10.3390/medicina62091721/s1, Supplementary Table S1, patient groups according to UTI-compatible symptoms and urine culture results; Supplementary Table S2, secondary (exploratory) analyses according to other OAD classes; Supplementary Table S3, distribution of microorganisms among patients with significant urine culture positivity.
