# Supplementary material for: Association of SGLT2 Inhibitor Use with Glycosuria, Pyuria, Urinary Symptoms, and Significant Urine Culture Positivity in Adults with Type 2 Diabetes: A Prospective Cross-Sectional Study
Source: Medicina (Kaunas). 2026 Sep 7;62(9):1721. doi: 10.3390/medicina62091721 (PMC13608954; doi:10.3390/medicina62091721)
Supplement: Supplementary file 1 [file medicina-62-01721-s001.zip › medicina-4519574-supplementary.pdf]

SUPPLEMENTARY MATERIALS

Association of SGLT2 Inhibitor Use with Glycosuria, Pyuria, Urinary Symptoms, and Significant Urine Culture Positivity in Adults with Type 2 Diabetes: A Prospective Cross-Sectional Study

Supplementary Table S1. Patient groups according to UTI-compatible symptoms and urine culture results.

Group	n (%)
UTI-compatible symptoms present + significant culture positive	9 (3.6)
UTI-compatible symptoms absent + significant culture positive	5 (2.0)
UTI-compatible symptoms present + culture negative	84 (33.5)
UTI-compatible symptoms absent + culture negative	119 (47.4)
Contaminated/uninterpretable culture	34 (13.5)

Percentages were calculated using the entire study population (n = 251) as the denominator.

Supplementary Table S2. Secondary (exploratory) analyses according to other OAD classes.

Drug class	Outcome	Nonusers, n/N (%)	Users, n/N (%)	Raw p-value	FDR-adjusted q-value
Metformin	Glycosuria	31/46 (67.4)	101/205 (49.3)	0.033	0.415
Metformin	Pyuria	8/46 (17.4)	55/205 (26.8)	0.258	0.718
Metformin	UTI-compatible symptoms	18/46 (39.1)	92/205 (44.9)	0.514	0.947
Metformin	Significant culture positivity	2/40 (5.0)	12/177 (6.8)	1.000	1.000
Metformin	Symptomatic culture positivity*	1/40 (2.5)	8/177 (4.5)	1.000	1.000
DPP-4 inhibitor	Glycosuria	90/189 (47.6)	42/62 (67.7)	0.008	0.201
DPP-4 inhibitor	Pyuria	45/189 (23.8)	18/62 (29.0)	0.404	0.947
DPP-4 inhibitor	UTI-compatible symptoms	89/189 (47.1)	21/62 (33.9)	0.078	0.647
DPP-4 inhibitor	Significant culture positivity	12/167 (7.2)	2/50 (4.0)	0.530	0.947
DPP-4 inhibitor	Symptomatic culture positivity*	7/167 (4.2)	2/50 (4.0)	1.000	1.000
Sulfonylurea	Glycosuria	122/237 (51.5)	10/14 (71.4)	0.176	0.718
Sulfonylurea	Pyuria	61/237 (25.7)	2/14 (14.3)	0.528	0.947
Sulfonylurea	UTI-compatible symptoms	104/237 (43.9)	6/14 (42.9)	1.000	1.000
Sulfonylurea	Significant culture positivity	13/203 (6.4)	1/14 (7.1)	1.000	1.000
Sulfonylurea	Symptomatic culture positivity*	8/203 (3.9)	1/14 (7.1)	0.458	0.947
Thiazolidinedione	Glycosuria	129/248 (52.0)	3/3 (100.0)	0.249	0.718
Thiazolidinedione	Pyuria	61/248 (24.6)	2/3 (66.7)	0.156	0.718
Thiazolidinedione	UTI-compatible symptoms	110/248 (44.4)	0/3 (0.0)	0.258	0.718
Thiazolidinedione	Significant culture positivity	14/214 (6.5)	0/3 (0.0)	1.000	1.000
Thiazolidinedione	Symptomatic culture positivity*	9/214 (4.2)	0/3 (0.0)	1.000	1.000
Acarbose	Glycosuria	132/249 (53.0)	0/2 (0.0)	0.224	0.718
Acarbose	Pyuria	63/249 (25.3)	0/2 (0.0)	1.000	1.000
Acarbose	UTI-compatible symptoms	109/249 (43.8)	1/2 (50.0)	1.000	1.000
Acarbose	Significant culture positivity	14/215 (6.5)	0/2 (0.0)	1.000	1.000
Acarbose	Symptomatic culture positivity*	9/215 (4.2)	0/2 (0.0)	1.000	1.000

OAD, oral antidiabetic drug; FDR, false discovery rate. Comparisons were performed using Fisher's exact test. Analyses of glycosuria, pyuria, and UTI-compatible symptoms included 251 patients; culture-based analyses included 217 patients after exclusion of contaminated cultures. *Symptomatic culture positivity was defined as the coexistence of UTI-compatible symptoms and significant culture positivity. Benjamini–Hochberg FDR correction was applied across all 25 exploratory OAD-class comparisons. No association remained statistically significant at $q < 0.05$. The numbers of thiazolidinedione and acarbose users were very low; estimates for these classes are particularly unstable.

Supplementary Table S3. Distribution of microorganisms among patients with significant urine culture positivity (n = 14).

Microorganism	n (%)
Escherichia coli	10 (71.4)
Klebsiella spp.	3 (21.4)
Candida albicans	1 (7.1)
